# Editorial: Evolution of Innate Immunity in Eukarya: Advances and Implications

**DOI:** 10.3389/fimmu.2022.879429

**Published:** 2022-04-20

**Authors:** Jean L. Scholz, Ioannis Eleftherianos, Bostjan Kobe

**Affiliations:** ^1^ Department of Pathology and Laboratory Medicine, University of Pennsylvania Perelman School of Medicine, Philadelphia, PA, United States; ^2^ Infection and Innate Immunity Laboratory, Department of Biological Sciences, The George Washington University, Washington DC, United States; ^3^ School of Chemistry and Molecular Biosciences, Australian Infectious Diseases Research Centre and Institute for Molecular Bioscience, The University of Queensland, Brisbane, QLD, Australia

**Keywords:** innate immunity, host-pathogen interactions, comparative immunology, immune signaling, cellular immunity

Immune networks are nearly as old as life itself. A rapidly-expanding body of evidence is revealing remarkable similarities in the recognition of non-self and in the attendant downstream signaling, across eukaryotic kingdoms. Studies are also revealing the likely drivers of the evolution of innate immune networks, both within eukaryotic cells and in the cellular ‘dialogue’ between the host and the corresponding commensal or pathogenic organisms. This Research Topic focuses on recent advances in our understanding of the origins of innate immunity, conditions that may have shaped its evolution in various species occupying different environments, and co-evolution between microbes and their host. It includes both reviews and primary research articles.

A large cluster of articles in this Research Topic comprises articles on the evolution of innate immune signaling mechanisms. Nimma et al. performed a comprehensive analysis of the structural features of assemblies formed by TIR (Toll/interleukin-1 receptor/resistance) domain-containing proteins, featuring in innate immunity pathways in animals and plants. They report that TIR-domain complexes can be classified into “scaffold” and “enzyme” assemblies, which play distinct roles in innate-immunity and cell-death signaling, respectively. The key enzymatic function featured by enzyme TIR domains is the cleavage of NAD^+^ (nicotinamide adenine dinucleotide). An important protein possessing such activity is SARM1, initially characterized as a Toll-like receptor (TLR) adaptor protein. In neurons, the catalytic activity of SARM1 TIR domain is induced upon axon injury and leads to axon destruction. DiAntonio et al. highlight the evolutionarily conserved enzymatic function of TIR-domain proteins as a new class of metabolic regulatory enzymes and discuss the regulatory and functional resemblance between SARM1 activation and bacterial toxin-antitoxin systems.

Ozakman et al. used *Drosophila* and its interaction with the insect parasitic nematodes *Heterorhabditis bacteriophora* as a model to present novel evidence on the function of activin and bone morphogenetic protein (BMP) signaling in the regulation of host immune responses to parasitic nematode infection. They show that BMP signaling activity modulates the DUOX/ROS response and that activin signaling activity modulates the antimicrobial peptide and melanization responses to nematode infection. These findings contribute to a better understanding of the evolution of the immune role of transforming growth factor β (TGF-β) signaling in invertebrates. Humoral and cellular immune signaling and function are well conserved in insects, but the timing of and duration of the response to bacterial infection is not well understood. Bruno et al. explored the immune reaction times in the black soldier fly *Hermetia illucens*, which comes into contact with a wide range of bacteria that live in decaying substrates. They find that cellular immune processes such as phagocytosis and encapsulation against Gram-negative and Gram-positive bacteria are activated faster than antimicrobial peptide activity in the hemolymph. This information can be used to improve mass-rearing practices, through enhancing insect immune capacity against microbial pathogens.

Comparing cellular immune processes between vertebrates and invertebrates can lead to the discovery of conserved mechanisms. Kokhanyuk et al. compared immune processes in coelomocytes in the earthworm *Eisenia andrei* and human macrophage-like cells. Using endocytosis inhibitors, to examine the engulfment mechanisms of *Escherichia coli* and *Staphylococcus aureus* bacteria, the authors show that although there were differences in intracellular signaling, bacterial internalization through actin-dependent phagocytosis in earthworm and human immune cells is well conserved. Comparative genomics analysis of immune molecules has the potential to reveal their potential conserved roles throughout evolution. Saco et al. performed a comparative genomics study of the key inflammatory cytokines in the interleukin-17 (IL-17) family across different phyla. Comparing multiple genomes from the Mediterranean mussel *Mytilus galloprovincialis*, they find 379 unique IL-17 sequences, which show similar patterns of expansion and variability to IL-17 sequences in other marine invertebrates. Functional validation of certain isoforms through experimental infection with *Vibrio* bacteria confirmed the conserved role of IL-17 in epithelial immune signaling. Li et al. look into the involvement of the newly described amphioxus (cephalochordate) apextrin C-terminal (ApeC)-containing proteins ACP3 and ACP5 in antimicrobial immune responses. They performed expression and microbial binding studies with recombinant proteins, to show that both ACPs bind and aggregate microbes. They exhibit binding specificity to microbial cell wall components, but lack microbial inhibitory activity, and ACP3 regulates the intracellular pathway involving TRAF6 and NF-κB. These findings have the potential to expand interest in understanding the immune role of ACPs in animals.

Recent work has led to an improved understanding of immune signaling and the evolution of immunity to pathogens in plants. Fang and Gu review the regulation of plant immunity by nuclear membrane proteins and highlight functional counterparts in animals. In particular, they discuss recent information on the significant participation of nucleoporins, nuclear transport receptors, and the nuclear lamina in the regulation of nuclear membrane-associated plant immunity. Also, on the topic of immunity in plants, another research article highlights the involvement of miRNAs in modulating the transcriptomic basis of the anti-fungal immune response in the poplar tree, *Marssonina brunnea*. Liao et al. use high-throughput sequencing, together with qPCR validation and functional approaches to identify key fungal-pathogen-responsive genes and miRNAs that take part in the poplar immune response to fungal infection, through regulating pathways that control diverse functions, including plant hormone signaling, antioxidant systems, and lignin biosynthesis.

The Research Topic also includes several articles analyzing the mutual evolutionary pressures between the host and the pathogen and the corresponding arms race. Tsu et al. highlight the evolutionary significance of interactions between viral proteases and the host. The authors summarize recent information suggesting that viral proteases from positive-sense single-stranded RNA genome have evolved to adapt to novel hosts and that this process continues to evolve. They posit that targeting essential host processes by viral proteases occurs in a virus-specific manner and this is indicated by the fact that viral proteases are typically directed against innate antiviral immune proteins. They further describe evolutionary conserved host signaling pathways that are known to detect viral protease activity and the consequences from this dynamic process. An important mechanism for the protection of host cells against viral infection is the necroptotic cell-death pathway. Águeda-Pinto et al. examine the molecular evolution of necroptosis in mammals and show that certain mammalian orders lost the necroptotic pathway during evolution. Their work suggests that disruption of necroptosis in mammalian lineages does not necessarily pose a disadvantage for their development, and that the corresponding naturally infecting poxviruses lost their ability to suppress this pathway. These findings demonstrate a co-evolutionary link between viral infection components and the immune signaling machinery in their hosts. Furthermore, genomic instability in the host may form an evolutionary mechanism to facilitate diversification of immune gene families. Barella Hudgell and Smith perform a detailed bioinformatic and phylogenetic examination of the immune-related *SpTransformer* (*SpTrf*) gene family in the genome of the purple sea urchin, *Strongylocentrotus purpuratus*, which reveals an additional cluster of genes. Analysis of the structural properties of this new gene cluster provides information on its evolutionary origin and offers clues for the type of genomic changes that took place through evolutionary history to expand the number of genes and their sequence variations in this gene family.

The manuscripts in this Research Topic present exciting findings and insights into the evolutionary basis of innate immune signaling and function in a wide range of eukaryotes. This information helps us clarify the fundamental processes that fine-tune the interplay between pathogenic microbes, their effector molecules, and the activity of host defense mechanisms. This is critical knowledge for designing novel means for the genetic manipulation of innate immune cells to combat the emergence of infectious diseases. We thank all the authors, reviewers and editors for their contributions to this collection.

## Author Contributions

All authors contributed to the article and approved the submitted version.

## Funding

The research in the authors’ laboratories was funded by the National Science Foundation (Award Number 2019869) to IE, the National Health and Medical Research Council (NHMRC) (Project Grant 1160570) and the Australian Research Council (ARC) (Discovery Projects DP190102526, DP220102832 and Laureate Fellowship FL180100109) to BK.

## Conflict of Interest

The authors declare that the research was conducted in the absence of any commercial or financial relationships that could be construed as a potential conflict of interest.

## Publisher’s Note

All claims expressed in this article are solely those of the authors and do not necessarily represent those of their affiliated organizations, or those of the publisher, the editors and the reviewers. Any product that may be evaluated in this article, or claim that may be made by its manufacturer, is not guaranteed or endorsed by the publisher.

